# Longitudinal Investigations of Autoregressive Cross-Lagged Path Models Among Internet Use, Executive Function Problems, and Maternal Control in Young Korean Children

**DOI:** 10.3389/fpsyt.2022.846995

**Published:** 2022-05-17

**Authors:** Hana Song

**Affiliations:** Department of Child Psychology and Education/Social Innovation Convergence, Sungkyunkwan University, Seoul, South Korea

**Keywords:** Internet use, executive function problems, young children, cross-lagged path model, maternal control

## Abstract

Excessive Internet use is related to behavioral and cognitive dysfunctions, but little is known about the relationship between them in young elementary school children. This study examined the longitudinal relationship between children's Internet use for entertainment purposes, executive function problems, and maternal control. Differences by children's Internet addiction status in their associations were also examined. Data from Wave 9 (2016) to Wave 11 (2018) of 1,463 children and mothers who participated in the Panel Study on Korean Children (PSKC) were used. The children's ages were 8 (grade 2), 9 (grade 3), and 10 years (grade 4) at Waves 9, 10, and 11, respectively. Associations between the variables were analyzed using autoregressive cross-lagged model estimations and multi-group analysis. The results showed the longitudinal stability of each of children's Internet use, executive function problems, and maternal control over the 3 years. Mutual associations between maternal control and children's Internet use were found especially in the low-risk group. In addition, children's executive function problems positively predicted Internet use, and negative associations from executive function problems to maternal control were significant over the years. However, some of these associations were significant only in the high-risk group. Discussions have focused on the protective role of maternal control and cognitive intervention, which could reduce children's excessive Internet use.

## Introduction

Excessive Internet use by young children has been a social issue in South Korea for many years. These days, children use the Internet almost every day for learning, interpersonal interaction, and entertainment (1, 2). However, according to a report by the Korean National Information Society Agency in 2020 (3), approximately one-fifth of children who have a mobile device are at risk for Internet addiction. Compared with children who use the Internet in an appropriate and controllable way for their age, children with excessive Internet use experienced attention problems and poor academic achievement (4), self-control deficiency (5), and interpersonal conflicts with parents and peers (6, 7). Although it is evident from neurophysiological studies that excessive Internet use undermines the cognitive development of young children (8, 9), executive function problems caused by excessive Internet use have received relatively little attention.

Executive function comprises a set of components that are necessarily involved in cognitive and behavioral control and problem solving, such as working memory and attentive control, inhibition and shifting, planning and organizing, and mental reasoning (10–12). Lack of executive functioning is often related to impulse control and compulsive disorders, as well as emotional dysfunction and carelessness (13–15). Although it has been reported that children with Internet addiction tend to show lower attention and executive function than non-addicted children do (16), the exploration of the relationship between Internet addiction and executive function needs to be further expanded by considering the following.

First, it is difficult to define “excessive Internet usage” by children resulting in executive function problems. It is also difficult to prove that normal Internet usage by children does not cause executive function problems and that only Internet addiction results in such problems. In particular, researchers (17, 18) have argued that the content viewed and the purpose of using the Internet might be a major concern, rather than the time spent using the internet. They have also suggested that children's unsupervised Internet use for sensation-seeking or for the compensation of psychosocial problems remains an issue regardless of time online. Parents and educators may believe that supervised Internet use by children will prevent excessive usage. However, in very young infants, inappropriate screen media exposure can adversely affect brain development (19). Thus, it is essential to monitor children's Internet usage for undesirable pleasure-seeking at all times, regardless of whether the children are addicted or not addicted to Internet use.

It would be noted that it is more common for mothers to care for children in Korea, and <10% of fathers substantially participated in childrearing in contemporary Korean society (20, 21). Although children's excessive Internet use is a concern for both parents, it would be more reasonable to believe that children's Internet usage is monitored and controlled mostly by their mothers. Maternal control was therefore the focus of this study.

Second, the direction of influence between excessive Internet use and executive function problems is not well understood. It is unclear whether one variable influences the other, or whether the two variables influence each other. For example, children at high risk for Internet addiction do not always show problems across all subcomponents of executive function, even though it is true that children with excessive Internet use report difficulty in inhibiting their needs when doing something or switching to something else (22). By contrast, not all children with a lack of attention and behavioral control display excessive Internet use (23). Therefore, the interaction between children's Internet use and executive function problems needs to be examined in both directions.

Third, since children's cognitive and self-regulatory abilities increase with age (24, 25), it is important to verify the longitudinal stability and changes in children's Internet use, executive function problems, and the relationship between the two. It is noteworthy that each subcomponent of executive function develops at different rates during childhood (26–29). While children's inhibitory control begins to develop in early childhood and continues to develop until adolescence or adulthood (26, 27, 30), cognitive flexibility and organizing skills develop more saliently during middle childhood and adolescence (31). Nevertheless, since the proportion of adolescents who are addicted to the Internet and have underlying cognitive problems is higher than that of young children (3), research on Internet addiction has much focused on the adolescent population. Relative to the large amount of data available on Internet usage and executive function problems in adolescents, little is known about the same in primary school children. Thus, emphasis must be placed on the longitudinal causal interaction of these variables among school-age children.

Fourth, supervised Internet use by parents is considered key to protecting children from Internet addiction, and its importance is well documented across studies (17, 32, 33). With regard to the Internet, it is known that parental monitoring and support lead to outcomes in children that are more positive (34–36). Children, whose parents set clear rules about and consistently monitored Internet use, had a lower risk for Internet addiction. By contrast, children of parents who did not spend much time monitoring Internet use for double-income or other reasons were at high risk for Internet addiction (34, 36). However, the relationship between parental control and excessive Internet use remains controversial. Although parental control is a protective factor for children's Internet use, extremely strict and harsh parental control affects children's mental health causing problems such as depression, which can be related to excessive Internet use (37, 38). In addition, the fact that parent-child interactions vary as children grow needs to be taken into consideration (39). It is reasonable to expect that the association between parental control and children's Internet use changes according to their development. Moreover, parental control has not been examined in relation to children's executive function problems. There is little evidence that parental control decreases or increases executive function problems in children. Parents are likely to strictly control their children when they exhibit impulsive behaviors or emotional dysregulation, which can be due to executive function problems. However, the dynamic relations between parental control, children's executive function problems, and Internet use have not been well investigated. It is unclear whether one variable is a protective or risk factor for the other, or whether variables interact independently with each other.

**Figure 1:**
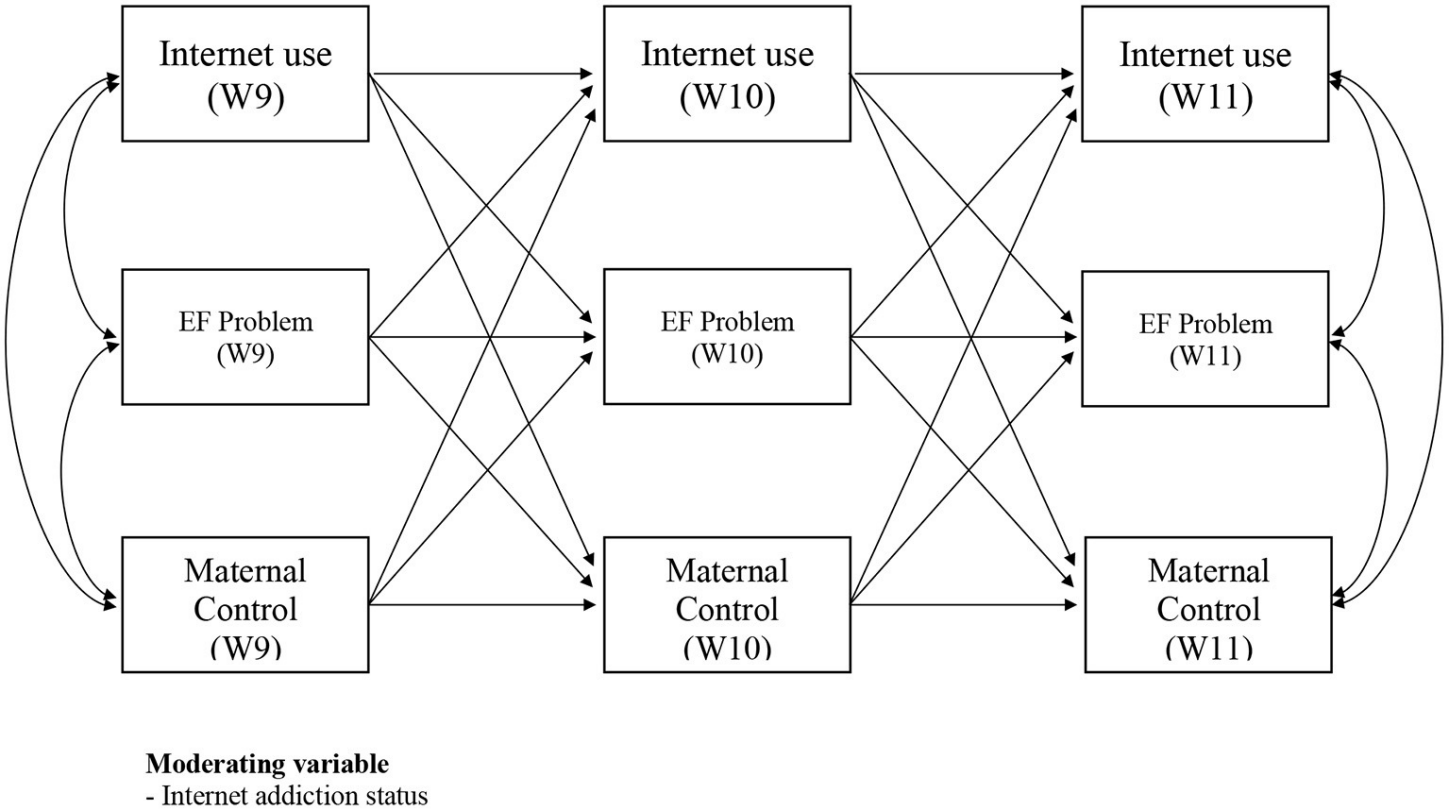
Hypothetical model (*N* = 1,463). EF, Executive Function.

On the basis of the four issues discussed above, this study aimed to examine the longitudinal relationship between children's Internet use, executive function problems, and maternal control in school-age children. For this purpose, the hypothesized model in Figure 1 was built. The first aim was to examine the longitudinal stability of three study variables (children's Internet use, executive function problems, and maternal control). A longitudinal study for at least three waves is required to determine causality and to test mediation effects and reciprocal associations among variables (40, 41). Consistent with previous findings (42, 43), I hypothesized that each of the three variables would remain stable, as children developed from Wave 9 to Wave 11. Although the previous studies have suggested that problematic Internet use in childhood and adolescence could last for years, it is not well known whether children's Internet use and executive function problems in lower grades of primary school generally continue or change with development. In addition, the developmental continuity and discontinuity of maternal control are not yet clearly understood, especially in relation to children's Internet use and executive function problems. Therefore, this study first hypothesized the stability of each of children's Internet use, executive function problems, and maternal control in childhood and tested whether a value at one point in time would be predicted by the future value of the same series.

The second aim was to examine longitudinal directionality between the three research variables. Previous findings indicated that time of Internet use positively predicted difficulty in attention and cognition processes (4, 16) and self-control (5, 44). Consistent with previous findings, I hypothesized that children's excessive use of the Internet would positively predict executive function problems. Further, I aimed to examine the longitudinal influences between maternal control and children's executive function problems. I hypothesized mutual influences between maternal control and children's executive function problems across time.

Finally, this study aimed to examine differences in the Internet addiction status (low-risk vs. high-risk) in the longitudinal relationship between children's Internet use, executive function problems, and maternal control. It is important to note that children's use of the Internet is not always a problem, and that severe dysfunction in cognition and behaviors is observed mostly in children with severe Internet addiction. In particular, clinical attention is mainly given to children who have shown Internet addiction for years, not to those who temporarily overuse the Internet. Therefore, this study classified children who showed excessive Internet use during all three waves as a high-risk group, and hypothesized that the longitudinal associations between the three variables would differ between children in the high-risk group and those who were not. Although it is expected that the association of children's executive function problems with other variables would be salient in the high-risk group, examination of Internet addiction status as a moderating variable was exploratory without hypothesizing a specific direction or pattern.

## Materials and Methods

### Participants

This study used data from Wave 9 (2016) to Wave 11 (2018) of 1,463 children and parents who participated in the Panel Study on Korean Children (PSKC). The PSKC is a nationwide longitudinal study conducted by the Korea Institute of Child Care and Education (KICCE), followed by developmental outcomes, family mental health, school adjustment, and other variables of 2,150 children and families since 2008 (at childbirth). The participants were recruited using a stratified multi-stage sampling method. First, 30 hospitals were recruited nationally. Among the families of newborns in these hospitals, 2,150 agreed to participate in the initial study, and continued to participate in annual follow-ups. Data were obtained from the children, parents, and teachers using questionnaires, web-based interviews, observations, and tests. The annual sample attrition from 2008 (Wave 1) to 2016 (Wave 9) was approximately 3 % on average. Of 1,556 families, data from 93 families, who were not present in two or more rounds of the survey on the research variables from Wave 9 to Wave 11, were excluded. Children and mothers who were excluded were compared with 1,463 participants, who remained in this study, but no critical differences were found. The excluded cases reported a lower percentage of college education (χ^2^ (1) = 7.99, *p* < 0.01), but no significant difference was found in family economic status (χ^2^ (1) = 0.59, *p* = 0.44). Of the 1,463 families included in this study, children's ages were 8 (grade 2), 9 (grade 3), and 10 years (grade 4) at Wave 9, 10, and 11, respectively, and about 51 % (*n* = 748) were boys, and 49 % (*n* = 715) were girls. The mothers' mean age was 31.21 years (SD = 3.65), and 70.8 % of the mothers graduated from college at the initial wave. Only 3.6 % (*n* = 49) of the families received financial assistance from the government.

### Measures

#### Children's Executive Function Problems

Children's executive function problems were measured using the original Korean version of the Executive Function Difficulty Screening Questionnaire (EFDSQ) (45). The EFDSQ consists of 40 Korean items to assess difficulties in planning-organizing, behavior control, emotional control, and attention-concentration of executive function. Mothers rated each item using a three-point scale, from *never* (1) to *always* (3), to indicate how often their child displayed a target behavior during six months. The total sum of the scores ranged from 40 to 120. A high score indicated high executive function problems. Cronbach's alphas for this scale were 0.94, 0.95, and 0.95, for Wave 9, 10, and 11, respectively.

#### Maternal Control

Maternal control was measured using four sub-items of the revised Korean version of the Inventory for Parenting Behavior (46). Mothers rated the extent to which they agreed with each of the four statements on parental monitoring and control children (e.g., “I know where my child is, and who my child is with”; “I know what my child does when he or she is alone”; “I know how to reach my child and keep in contact with my child.”) on a five-point scale ranging from *not true* (1) to *very true* (5). The total sum of the scores range from 4 to 20. A high score indicates a high level of maternal control. The internal consistency of this scale was satisfactory, with Cronbach's alphas for this scale were 0.79, 0.81, and 0.81 for Waves 9, 10, and 11, respectively.

#### Children's Internet Use

Caregivers, mostly mothers, reported the amount of times their children spent on the Internet through computers or smartphones each day, and the purpose of Internet use for the three waves. Children's Internet use was computed as the total time of Internet use multiplied by the percentage of nonacademic use, such as games, TV, social network service (SNS), and web surfing. A high score indicates a high level of Internet use for entertainment purposes.

#### Children's Internet Addiction Status (Moderator Variable)

Children were divided into two groups based on two indices: low-risk (normal) and high-risk for Internet addiction. First, children who used the Internet for more than 2.5 h every day from Wave 9 to Wave 11 were identified by maternal report (e.g., “How much time does your child spend on the Internet on an average day?”). In addition, at Wave 11, children were assessed using 15 items of a revised Korean version of the diagnostic scale for Internet overdependence (K-scale) developed by the National Information Society Agency (47). Items on the K-scale describe a child's behavioral problems related to Internet use, and mothers rated each item on how likely their children were to show such behaviors, using a four-point scale from *not likely at all* (1) to *very likely* (4). On the K-scale, a total score of 30 or above indicates problematic Internet use. Cronbach's alpha for the K-scale was 0.86. Children in the high-risk group were characterized by excessive use of and problematic dependence on the Internet every day, whereas low-risk children did not show these problems.

### Data Analysis Plan

SPSS version 27.0 was used for the analyses of the descriptive statistics and bivariate correlations, and AMOS was used for the hypothesized cross-lagged panel model (CLPM) analysis. A CLPM is the statistical model which allows the examination of longitudinal sequential associations between variables (48). Typically, the CLPM includes three parameters: (a) autoregressive paths, (b) cross-lagged paths, and (c) contemporaneous correlations. The autoregressive paths of the model, in which repeatedly assessed variables are regressed on their immediate prior values, are used to test (rank-order) stability within the same variables. For example, the variance in maternal control at Wave 10 and 11 was derived from the maternal control at Waves 9 and 10. The cross-lagged paths enable testing of longitudinal directionality between different variables. For example, variance in Internet use at Wave 10 was derived from the variance of executive function problems and maternal control at Wave 9. The contemporaneous correlations between the three parallel-assessed variables were also included in the model.

First, the data of 1,463 children included missing values for executive function problems (*n* = 168), maternal control (*n* = 157), and Internet use (*n* = 101) from Wave 9 to Wave 11. Regression imputation was applied for the missing values. A CLPM analysis with Maximum Likelihood Estimation (MLE) was performed. Model fit was assessed using Comparative Fit Index (CFI), Tucker–Lewis Index (TLI), and Root Mean Square Error of Approximation (RMSEA). CFI and TLI ≥ 0.90 and RMSEA < 0.08 indicate an acceptable fit (49). Standardized path coefficients and R-values were reported as effect sizes [r-matrix; (50)]. Further, improper parameter estimation was examined (e.g., negative variances /standardized values over 1.0). Following the suggestion of previous studies (51, 52), those improper parameters were fixed to zero.

Differences between high-risk and low-risk groups in the CLPM were then tested by multi-group analysis to estimates moderating effects of Internet addiction status. A nested model comparison test was performed to examine whether the estimated coefficients of CLPM varied depending on Internet addiction status. For nested model comparisons, a chi-square test was used. A significant *p*-value (level of 0.05) of the chi-square indicates that the coefficients in one group are different from those in another. Separate estimates of the predictive paths for the two groups were compared.

## Results

### Preliminary Analysis

Table 1 shows zero-order correlations and descriptive statistics for the total sample (a), and by Internet addiction status (b). The numbers above and below diagonal indicate the correlations for the high-risk and the low-risk group, respectively. All bivariate correlations within each of the three research variables were statistically significant. Children's Internet use was positively and negatively correlated with executive function problems and maternal control, respectively, across the three waves as well as within the same wave. However, in the split by Internet addiction status, significant negative correlations between children's Internet use and maternal control were found more in the low-risk group. For descriptive statistics, children at high risk for Internet addiction reported more executive function problems than those in the low-risk group at Wave 9 and Wave 11 (*t* = −3.06–−2.07, *p* < 0.05, *d* = 0.15–0.22). In addition, maternal control was significantly higher in the low-risk group than in the high-risk group at Wave 10 and Wave 11 (*t* = 2.05–3.32, *p* < 0.001, *d* = 0.16–0.26).

**Table 1:** Descriptive statistics and bivariate correlations comparing high-risk and low-risk groups.

	**1**	**2**	**3**	**4**	**5**	**6**	**7**	**8**	**9**	***M***	***SD***
(a)											
1.Internet use, 9	–									0.84	0.7
2.Internet use, 10	0.30^***^	–								1.19	0.81
3.Internet use, 11	0.27^***^	0.38^***^	–							1.5	0.93
4.EFP, 9	0.06^*^	0.07^*^	0.11^***^	–						58.71	12.55
5.EFP, 10	0.05^*^	0.05	0.07^*^	0.68^***^	–					59.1	12.68
6.EFP, 11	0.03	0.04	0.12^***^	0.62^***^	0.69^***^	–				57.51	12.51
7.MC, 9	−0.06^*^	−0.08^**^	−0.11^***^	−0.14^***^	−0.1^***^	−0.12^***^	–			19.36	1.34
8.MC, 10	−0.04	−0.06^*^	−0.09^**^	−0.09^***^	−0.14^***^	−0.11^***^	−0.19^***^	–		19.09	2.46
9.MC, 11	−0.01	−0.08^**^	−0.11^***^	−0.14^***^	−0.13^***^	−0.15^***^	0.21^***^	0.25^***^	–	18.58	1.71
											
(b)											
1.Internet use, 9	–	0.26^***^	−0.01	0.05	0.12	0.02	0.03	0.02	0.17^*^	1.21	0.98
2.Internet use, 10	0.26^***^	–	0.13	0.09	0.09	0.06	−0.11	−0.18	−0.02	1.75	1.05
3.Internet use, 11	0.24^***^	0.31^***^	–	0.24^***^	0.09	0.21^**^	−0.24^**^	−0.08	−0.11	3.27	0.76
4.EFP, 9	0.05	0.05	0.07^*^	–	0.65^***^	0.67^***^	−0.15^*^	0.08	−0.17^*^	60.4	13.42
5.EFP, 10	0.03	0.03	0.05	0.68^***^	–	0.69^***^	−0.17^*^	0.03	−0.14^*^	60.25	12.57
6.EFP, 11	0.01	0.02	0.06^*^	0.61^***^	0.69^***^	–	−0.21^**^	0.03	−0.15^*^	60	13.04
7.MC, 9	−0.08^**^	−0.06^*^	−0.09^**^	−0.14^**^	−0.08^**^	−0.1^**^	–	0.26^***^	0.25^***^	19.23	1.61
8.MC, 10	−0.05	−0.03	−0.08^**^	−0.12^***^	−0.17^***^	−0.12^***^	0.1^***^	–	0.34^***^	18.76	2.31
9.MC, 11	−0.04	−0.07^*^	−0.03	−0.13^***^	−0.12^***^	−0.14^***^	0.2^***^	0.23^***^	–	18.17	1.97
*M*	0.79	1.1	1.22	58.44	58.92	57.11	19.38	19.14	18.65	–	–
*SD*	0.63	0.73	0.58	12.39	12.7	12.38	1.29	2.48	1.65	–	–

*N = 1,463; M, mean; SD, standard deviation; Correlations above the diagonal represent the scores for high-risk group, scores below the diagonal represent the scores for low-risk group; EFP, Executive Function Problem; MC, Maternal Control*.

* *p < 0.05*

** *p < 0.01*

*** *p < 0.001*.

### Longitudinal Stability and Directionality (Aim 1 and 2)

**Figure 2:**
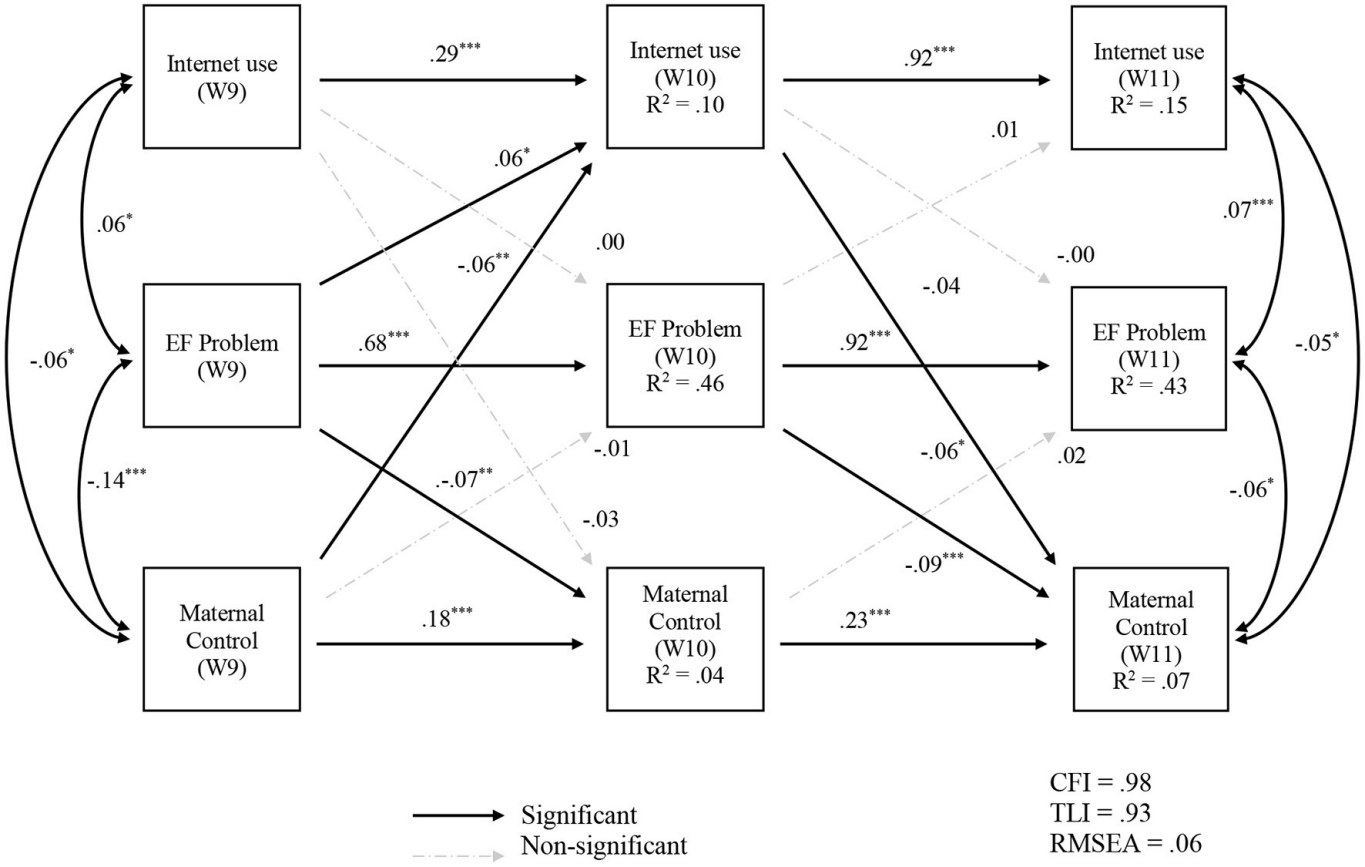
Autoregressive cross-lagged path model of Internet use, executive function problem, and maternal control for total sample (*N* = 1,463). Standardized regression coefficients were shown. EF = Executive Function. *p < 0.05, **p < 0.01, ***p < 0.001.

The results of the CLPM analysis examining the longitudinal stability and directionality between variables with the total sample are presented in Figure 2. This model had an acceptable fit, CFI = 0.98, TLI = 0.93, and RMSEA = 0.06. The results indicate that all autoregressive paths across the three waves were significant. For example, children's Internet use at Wave 9 and Wave 10 predicted children's Internet use at Wave 10 (β = 0.29*, p* < 0.001) and Wave 11 (β = 0.92, *p* < 0.001), respectively. The children's executive function problems (β = 0.68–0.92, *p* < 0.001) and maternal control (β = 0.18–0.23, *p* < 0.001) at Wave 10 and 11 were also predicted by the same variable in the previous waves. With regard to the cross-lagged paths, children's executive function problems at Wave 9 significantly predicted children's Internet use (β = 0.06, *p* < 0.05) and maternal control (β = −0.07, *p* < 0.01) at Wave 10. Maternal control at Wave 9 negatively predicted children's Internet use at Wave 10 (β = −0.06, *p* < 0.01). In addition, maternal control at Wave 11 was negatively predicted by children's Internet use (β = −0.06, *p* < 0.05) and executive function problems (β = −0.09, *p* < 0.001) at Wave 10. The results also indicate that contemporaneous correlations (r = −0.14–0.07, *p* < 0.05) among the study variables were significant across the waves.

### Moderation Effects of Internet Addiction Status (Aim 3)

A multi-group analysis for the CLPM split by Internet addiction status (low-risk vs. high-risk) was also performed, and the results of the chi-square difference test for nested model comparison indicated significant moderating effects of the Internet addiction status, χ^2^ = 45.70 (*df* = 18, *p* < 0.001). Table 2 displays the standardized path coefficients for low-risk and high-risk groups. As shown in Table 2, most of the autoregressive paths across the three waves were significant in both groups. Children's executive function problem at Wave 10 was also negatively associated with maternal control at Wave 11 in both groups (see corresponding coefficients in Table 2). However, mutual associations between children's Internet use and maternal control between Wave 10 and Wave 11 were significant only in the low-risk group (β = −0.06, *p* < 0.05), but not in the high-risk group. In addition, the paths from maternal control at Wave 9 to children's executive function problems at Wave 10 (β = −0.11, *p* < 0.01), and from children's executive function problems at Wave 10 to Internet use at Wave 11 (β = 0.34, *p* < 0.01) were significant only in the high-risk group.

**Table 2:** Standardized path coefficients in the low-risk and the high-risk group.

			**Low-risk (***N*** = 1,260)**		**High-risk (***N =*** 203)**	
			**β**	***p***	**β**	***p***
	Autoregressive paths					
Internet use, 9	→	Internet use, 10	0.25	<0.001	0.26	<0.001
EFP, 9	→	EFP, 10	0.69	<0.001	0.62	<0.001
MC, 9	→	MC, 10	0.16	<0.001	0.28	<0.001
Internet use, 10	→	Internet use, 11	0.31	<0.001	0.09	0.19
EFP, 10	→	EFP, 11	0.69	<0.001	0.69	<0.001
MC, 10	→	MC, 11	0.21	<0.001	0.35	<0.001
	Cross-lagged paths					
Internet use, 9	→	EFP, 10	−0.00	0.91	0.08	0.12
Internet use, 9	→	MC, 10	−0.03	0.27	0.01	0.87
EFP, 9	→	Internet use, 10	0.03	0.33	0.06	0.36
EFP, 9	→	MC, 10	−0.10	<0.001	0.12	0.08
MC, 9	→	Internet use, 10	−0.04	0.15	−0.11	0.12
MC, 9	→	EFP, 10	0.01	0.55	−0.11	<0.05
Internet use, 10	→	EFP, 11	−0.01	0.61	0.00	0.93
Internet use, 10	→	MC, 11	−0.06	<0.05	0.07	0.26
EFP, 10	→	Internet use, 11	0.05	0.17	0.34	<0.01
EFP, 10	→	MC, 11	−0.09	<0.01	−0.15	<0.05
MC, 10	→	Internet use, 11	−0.06	<0.05	−0.08	0.26
MC, 10	→	EFP, 11	−0.01	0.62	0.01	0.85

*EFP, Executive Function Problem; MC, Maternal Control*.

## Discussion

### Discussion of Research Findings

This study examined the longitudinal stability, directionality, and cross-variable influence of children's Internet use, executive function problems, and maternal control using panel data of children aged 8–10 years. Differences in the associations between the three research variables caused by Internet addiction status were also examined using multi-group analysis. Hypothesized autoregressive cross-lagged paths were tested with the total sample, and then with each of the low-risk and high-risk groups for Internet addiction. The major findings of this study are as follows.

As expected in the first hypothesis, the results of this study provide evidence of longitudinal stability of the three research variables. Maternal control and children's executive function problems tended to be stable in the lower grades of elementary school years. In particular, children's Internet usage continued in line with development during this period. For example, Internet usage time for 10-year-old children can be predicted by usage time a year before. Excessive Internet use and general Internet usage at a younger age are likely to continue. For example, children who used the Internet for more than 2.5 h per day at 8 years showed similar usage at 9 years. These findings are somewhat consistent with a previous study by Shek and Yu (43), in which addictive Internet use lasted for 2 years in adolescence. In a follow-up study by these researchers (42), the number of adolescents identified as Internet addicted decreased slightly in the third year, but there was no noticeable change in the prevalence of Internet addiction among adolescents with negative family environments. The current study expends these studies by findings a similar stability of Internet use in young elementary school children. It should be noted that the current study counted only Internet use for entertainment purposes such as games and SNS, and not for academic purposes. In other words, the results suggest that children's excessive pleasure-seeking through Internet games is likely to continue if there is no intervention. Therefore, it may be necessary to monitor not only patterns and times but also the purposes of Internet use as early as possible to prevent Internet addiction in later development.

Secondly, this study hypothesized cross-lagged causality and found significant cross-variable relationship between children's Internet use, executive function problems, and maternal control with the total sample. Specifically, there were mutually negative associations between maternal control and children's Internet use across waves. This finding shows that maternal control consistently contributed to reducing children's time spent using the Internet for entertainment purposes. This result is consistent with the findings of other studies in which maternal control or monitoring was a protective factor for children's excessive Internet use (34, 36). It may be true that many parents are being challenged and struggling to restrict their children's access to and use of harmful Internet games and web surfing in their daily lives. Nevertheless, parents are believed to be an important component in keeping children from excessive Internet use.

With regard to cross-relations between children's Internet use and executive function problems, i hypothesized bidirectional interactions between the two. However, the causal direction was found only in the pathway from children's executive function problems at Wave 9 to Internet use at Wave 10. This result is consistent with the findings of previous studies, in which children with low attention and impaired cognitive functioning were more likely to be addicted to the Internet (23, 53). In particular, Dovis and colleagues showed that training executive functioning enhanced working memory and inhibitory control of children with ADHD (54). Considering these previous findings, the results of the current study suggest that interventions for children with problematic Internet use could be most effective when various training programs to enhance their executive functioning are included. Although excessive Internet use is known to damage brain function, including inhibitory control and neural connectivity (55, 56), a similar finding was not reported in the current study. Nevertheless, it would not be appropriate if we hastily concluded that Internet addiction does not affect children's executive function. One possible explanation for this finding may be related to the period of longitudinal data. This study analyzed data from 3 years of childhood, but it is difficult to determine how long it takes for excessive Internet use to lead to damage to executive functioning. A three-year period is probably not long enough to observe such a phenomenon. It may be necessary to trace children's Internet use and executive function for a longer period of time.

In addition, associations between children's executive function problems and maternal control were significant in only one direction in the total sample. Children's executive function problems negatively predicted maternal control. However, there is no plausible explanation for this result. It is only assumed that maternal control may not be frequently delivered to children when the children show cognition-related problems due to a lack of central executive functioning. Parenting strategies other than maternal control may be more appropriate for dealing with children's executive function problems. Further investigation is required to determine this possibility.

As expected in the third hypothesis, differences between the low- and high-risk groups in the CLPM were found. With regard to Internet addiction status, mutual associations between children's Internet use and maternal control were found only in the low-risk group. None of the same paths were significant in the high-risk group. In contrast to previous studies (57, 58) suggesting that parental control considerably mitigates the risk of adolescents' Internet addiction, the protective role of parents of school-aged children who have been using the Internet excessively for years seems to be lower. However, it is not clear whether this is due to Internet addiction status, child age, or an interaction between them, because the influence of maternal control on children's Internet use was significant for the whole sample in the current study. Nevertheless, this study suggests that maternal control may not be fully effective, especially for young elementary school children at high risk for Internet addiction.

Children's executive function problems were positively associated with their Internet use only in the high-risk group. As mentioned above, low executive functioning in children is a risk factor for Internet addiction. In addition, a noticeable group difference was revealed in the association between maternal control and children's executive function problems. Contrary to the direction of these two variables in the total sample, maternal control negatively affected children's executive function problems only in the high-risk group. Executive function problems often involve impulsiveness and behavioral dysregulation, which can cause parents to be stressed and agitated (59). Thus, it may be common if parents try to control their children's behavioral problems caused by a lack of cognitive function. However, it must be noted that the influence of maternal control on children's Internet use was less significant in the high-risk group in the current study. Executive function problems of children at a high risk for Internet addiction were associated both with their Internet use and maternal control to some extent. The dynamic causal interactions between the three research variables remain somewhat unclear. Future studies are needed to determine these relationships in more detail.

In light of the findings discussed here, this study has implications for interventions for problematic Internet use. Most importantly, controlled and supervised Internet use by mothers was found to be a protective factor against excessive Internet use in young school-age children. However, mothers' involvement in controlling Internet usage time at home may not be fully effective for children who need therapeutic intervention for Internet addiction. It is important to note that there was a causal association between maternal control and children's executive function problems, especially in the high-risk group in this study. Therefore, combining cognitive training with maternal involvement to increase executive functioning may be an effective strategy for the treatment of Internet addiction in young children. Moreover, identifying children's executive function problems as early as possible may be an important component for prevention and intervention for Internet addiction.

### Limitations and Suggestions for Future Research

This study is significant in that it conducted a longitudinal investigation to determine causal directions based on a considerably large number of samples in typical and Internet-addicted groups of children. Furthermore, it explains children's Internet usage and related variables during lower grades in primary school, a relatively less studied age group. Nevertheless, it also has certain limitations that should be addressed for future studies. First, although lower executive functioning was found to be a possible cause of problematic Internet use, it is unclear whether this relationship can be applied to clinical settings. For instance, the levels and patterns of executive function problems in children with ADHD or other cognitive impairments differ from those in healthy children. Therefore, future studies should consider children with cognitive mental disorders and examine their neurological impairments in executive functioning. Second, this study was limited in controlling or analyzing demographics because the variations in these variables were not fully extensive. Overall, the participants in this study represented a middle-class population. However, many previous studies on Internet addiction and maternal behaviors have reported differences in children's gender and family economic status. Therefore, the potential influence of demographics should be controlled or analyzed in future studies. Third, despite using a representative sample, this study is not completely free from the limitations of panel data, such as endogeneity or autocorrelations. In addition, children's Internet use and executive function problems were measured only on the basis of mothers' reports. Although the scales had a satisfactory level of reliability, there may be a gap in the children's behaviors in mothers' reports and those in real settings. Therefore, measures that include observations need to be taken to address the issue of ecological validity in future studies. Fourth, the parental controls reported by fathers were not included in this study. Since it is more common for mothers or women to care for children in Korean families, fathers rarely monitor their children's Internet use during the daytime. However, children who show excessive Internet use may have conflicts with both their mothers and fathers. Most importantly, the discipline of fathers, which is more intermittent but more intense than that of mothers, is also likely to affect children's Internet use. Composite patterns of paternal and maternal control and their interactions should be considered in future studies. Finally, parental control in the dimension of general parenting also involves psychological ways of exacerbating children's negative emotional outcomes. Consequently, the follow-up of this study will examine the influence of both mothers and fathers on their children's Internet use at a more elaborated level of behavioral and psychological control.

## Data Availability Statement

Publicly available datasets were analyzed in this study. This data can be found here: https://panel.kicce.re.kr/pskc/module/rawDataManage/index.do?menu_idx=56.

## Ethics Statement

Ethical review and approval was not required for the study on human participants in accordance with the local legislation and institutional requirements. Written informed consent from the participants' legal guardian/next of kin was not required to participate in this study in accordance with the national legislation and the institutional requirements.

## Author Contributions

HS contributed entirely to all procedures for conceptualization, hypothesis, analysis, writing, and revision.

## Conflict of Interest

The author declares that the research was conducted in the absence of any commercial or financial relationships that could be construed as a potential conflict of interest.

## Publisher's Note

All claims expressed in this article are solely those of the authors and do not necessarily represent those of their affiliated organizations, or those of the publisher, the editors and the reviewers. Any product that may be evaluated in this article, or claim that may be made by its manufacturer, is not guaranteed or endorsed by the publisher.
